# A functional gene module identification algorithm in gene expression data based on genetic algorithm and gene ontology

**DOI:** 10.1186/s12864-023-09157-z

**Published:** 2023-02-17

**Authors:** Yan Zhang, Weiyu Shi, Yeqing Sun

**Affiliations:** 1 College of Environmental Science and Engineering, Dalian Maritime University, 116026 Dalian, Liaoning China; 2 College of Maritime Economics & Management, Dalian Maritime University, 116026 Dalian, Liaoning China

**Keywords:** Functional gene module, Overlapping gene module, Genetic algorithm, Gene ontology, Partitioning around medoids, Gene expression data

## Abstract

Since genes do not function individually, the gene module is considered an important tool for interpreting gene expression profiles. In order to consider both functional similarity and expression similarity in module identification, GMIGAGO, a functional Gene Module Identification algorithm based on Genetic Algorithm and Gene Ontology, was proposed in this work. GMIGAGO is an overlapping gene module identification algorithm, which mainly includes two stages: In the first stage (initial identification of gene modules), Improved Partitioning Around Medoids Based on Genetic Algorithm (PAM-GA) is used for the initial clustering on gene expression profiling, and traditional gene co-expression modules can be obtained. Only similarity of expression levels is considered at this stage. In the second stage (optimization of functional similarity within gene modules), Genetic Algorithm for Functional Similarity Optimization (FSO-GA) is used to optimize gene modules based on gene ontology, and functional similarity within gene modules can be improved. Without loss of generality, we compared GMIGAGO with state-of-the-art gene module identification methods on six gene expression datasets, and GMIGAGO identified the gene modules with the highest functional similarity (much higher than state-of-the-art algorithms). GMIGAGO was applied in BRCA, THCA, HNSC, COVID-19, Stem, and Radiation datasets, and it identified some interesting modules which performed important biological functions. The hub genes in these modules could be used as potential targets for diseases or radiation protection. In summary, GMIGAGO has excellent performance in mining molecular mechanisms, and it can also identify potential biomarkers for individual precision therapy.

**Supplementary Information:**

The online version contains supplementary material available at 10.1186/s12864-023-09157-z.

## Introduction

With the development and general application of high-throughput sequencing technology, a lot of gene expression data (RNA-Seq data, microarray data, etc.) have been accumulated in biological research. How to mine biological knowledge from these data effectively is an urgent problem to be solved. Many studies show that genes do not function individually, so the gene module is considered an useful tool for interpreting gene expression profiles [1–3]. Generally, a module is a group of co-expressed genes (genes with similar expressions) with similar functions [3, 4], which is mainly obtained through Gene Co-expression Network (GCN) analysis [5] currently, so it can also be called a co-expressed module. Nodes in GCN denote genes, and edges denote relationships between genes. Whether there is an edge between two genes is determined by the correlation between gene expression. Pearson correlation coefficient is the most commonly used measure, and nonlinear measures such as Spearman rank correlation coefficient and mutual information can also be used [6]. One of the most widely used pipelines in GCN analysis is “weighted gene co-expression network analysis (WGCNA)” [7, 8], the core of which is to use hierarchical clustering to identify gene co-expression modules. It can be further used for downstream tasks such as correlation analysis between modules and external indicators, and identification of hub genes. Recently, WGCNA has been applied in many research directions [9, 10].

Based on WGCNA, researchers have proposed many gene co-expression module identification algorithms. Zhang et al. introduced quasi-cliques detection technique and proposed a new method (lmQCM) to mine gene modules in weighted networks, which could obtain more functional and genetic information from expression data [11]. Dai et al. proposed csuWGCNA by combining signed and unsigned methods, and it improved the detection of negative correlation [12]. Hou et al. improved WGCNA by adjusting the hierarchical clustering while maintaining the advantages of dynamically cut trees, so that all genes could be assigned to modules with the highest average connectivity [13]. In the early stage, our team combined Newman and K-Means algorithms to propose a new method of gene module identification: GCNA-Kpca [4]. This algorithm took into account the role of modularity in the clustering process and could find the optimal membership module for each gene through multiple iterations. On the other hand, since a gene (or protein) is involved in multiple functions, overlapping gene modules (i.e. a gene can belong to one or more modules) are more reflective of the real biological system [14, 15]. Based on independent component analysis (ICA), principal component analysis (PCA), and independent principal component analysis (IPCA), Nguyen et al. proposed a method for the identification of overlapping gene co-expression modules: oCEM, which had good performance in detecting clinically relevant co-expression modules [3]. Camila et al. further extended WGCNA, using hierarchical link clustering to identify communities (modules) in co-expression networks, and identified 19 rice genes associated with salt stress [15]. The above methods had achieved good results in the identification of gene co-expression modules, but they only considered the similarity between gene expressions in the clustering process, while ignoring the functional similarity within the modules. The original purpose of gene module identification is to find some groups of genes with similar expressions and similar functions [3, 4], so how to satisfy these two conditions at the same time is a tough problem.

Gene functions are usually described qualitatively, so it is difficult to compare them directly [16]. Fortunately, the Gene Ontology (GO) project (http://www.geneontology.org/) provides structured, controlled vocabularies and classifications that cover several domains of molecular and cellular biology [17]. In general, which GO terms are overrepresented or underrepresented can be judged based on annotations. This process is called Gene Ontology Enrichment Analysis (GOEA), which can find the GO terms that are most relevant to a given gene module [18]. Further, we can calculate the similarity of significantly enriched GO terms to measure the functional similarity of genes in this module. At present, a popular similarity calculation strategy for GO terms is based on the Information Content (IC), which is to measure the importance of each term in the Directed Acyclic Graph (DAG) of GO [19]. And the similarity of the two GO terms depends on their respective IC, as well as the IC of their Lowest Common Ancestor (LCA) [16].

After determining the evaluation indicators of functional similarity, the identification of functional gene modules was transformed into a combinatorial optimization problem in this work. Namely, with the goal of increasing the functional similarity within modules, the membership modules for each gene (the modules to which each gene belongs) were continuously adjusted under certain rule constraints, and the Genetic Algorithm (GA) was used to optimize this process. GA is a bio-inspired meta-heuristic approach based on the concepts of biological evolution and survival of the fittest individuals [20]. Recently, GA has been used to solve various difficult optimization problems. Although GA may not obtain the optimal solution, it can provide better results within a reasonable time [21]. There are three main operators in GA: selection, crossover and mutation, out of which crossover is the most important operator [22]. In order to make GA converge faster, enough information needed to be inherited from elite individuals (individuals with better fitness). Therefore, we referred to [20] and used the elite crossover strategy in the selection operator. This strategy uses all individuals in a generation to cross with elite individual in turn, and elite individual can be replaced by one with better fitness at any time. GA using elite crossover strategy is called elite GA (EGA) [20]. Some studies have applied GA to the identification of gene modules. Toubiana et al. developed a GA to optimize gene modules by gradually increasing the correlation between traits and a subset of gene modules [23]. Novoa et al. proposed a multi-objective GA for identifying active modules in MUltiplex biological Networks, which optimized the density of interactions and the scores of the nodes [24]. However, GA was rarely applied to the optimization of functional similarity within gene modules.

**Fig.1:**
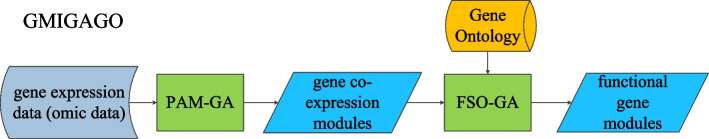
Flow-chart of GMIGAGO

We proposed GMIGAGO (Fig. 1), a functional Gene Module Identification algorithm based on Genetic Algorithm and Gene Ontology, considering both the expression similarity and function similarity within the modules. GMIGAGO is an overlapping gene module identification algorithm actually, which mainly includes two stages: In the first stage (initial identification of gene modules), Improved Partitioning Around Medoids Based on Genetic Algorithm (PAM-GA) is used for the initial clustering of gene expression data, and traditional gene co-expression modules, also called clusters, can be obtained. Only similarity of expression levels is considered at this stage. In the second stage (optimization of functional similarity within gene modules), Genetic Algorithm for Functional Similarity Optimization (FSO-GA) is used to optimize gene modules based on gene ontology. Finally, functional similarity within gene modules can be improved. Moreover, to verify the effectiveness of GMIGAGO in application, we carefully analyzed the gene modules in six datasets, including enrichment results analysis, association analysis between modules and clinical indicators, and identification of hub genes, etc. The results showed that the gene modules identified by GMIGAGO performed important biological functions, and the hub genes in the key modules could also be used as a potential therapeutic targets. The above results demonstrated that our functional gene modules had significant biological significance.

## Materials and methods

### Initial identification of gene modules

#### Partitioning around medoids

Partitioning Around Medoids (PAM) is a classic clustering method, which uses $k$ representative objects called medoids (chosen from the dataset) that serve as prototypes (centers) for the clusters instead of means. Therefore, one of the advantages of PAM is that it can use any custom distance [25]. The steps of the traditional PAM are as follows: (1) Input $n$ genes ${\{g}_{1},{g}_{2},\cdots ,{g}_{n}\}$ and cluster number $k$. (2) $k$ genes are randomly selected from the dataset as the initial medoid set (the K-Means +  + strategy is used to ensure that the initial medoids are sufficiently dispersed). (3) Assign each gene into the cluster represented by the medoid closest to it. (4) Calculate the sum of the distances (${d}_{sum}$) that all genes to the medoid of the cluster to which they belong. (5) Randomly update the medoid set and repeat (3) and (4) until the maximum number of iterations is reached. A new medoid set is accepted if it reduces ${d}_{sum}$.

Drawing on the method of gene co-expression network construction [26, 27], our distance between two genes was defined based on the Pearson correlation coefficient (Formula 1):

1 $$Dist=1-\frac{\sum_{i=1}^{m}({g}_{1i}-{\overline{g} }_{1})({g}_{2i}-{\overline{g} }_{2})}{\sqrt{\sum_{i=1}^{m}{({g}_{1i}-{\overline{g} }_{1})}^{2}}\sqrt{\sum_{i=1}^{m}{({g}_{2i}-{\overline{g} }_{2})}^{2}}}$$

where, ${g}_{1i}$ denotes the expression level of gene 1 in the ${i}_{th}$ sample, ${\overline{g} }_{1}$ denotes the mean expression level of gene 1 in all samples, and $m$ denotes the number of samples. We ran the PAM with a range of different values (from 8 to 20) of cluster number $k$, and the $k$ that optimized the Silhouette Score (Formula 2 ~ 3) was determined. The maximum number of iterations per run was set to 2000.

#### Improved Partitioning Around Medoids Based on Genetic Algorithm (PAM-GA)

In order to solve the problem that PAM searched medoids blindly and had difficulty in convergence, we proposed a new clustering algorithm: PAM-GA, as shown in Algorithm 1. Specifically, a GA is designed to search for medoids in PAM, replacing the process of randomly selecting medoids in traditional PAM (Algorithm 1, line 1 ~ 8): (1) Initialize the population (line 1). (2) Evaluate the fitness of each individual in the population according to the objective function, and select the elite individual (line 2 ~ 3). (3) Perform crossover and mutation operations to generate the next generation of population, and continue to iterate until the termination conditions are met (line 4 ~ 8). (4) Finally, clustering is performed based on the optimal medoid set. Namely, assign each gene into cluster represented by the medoid closest to it, and get the final clustering result (line 9).

**Figure:**
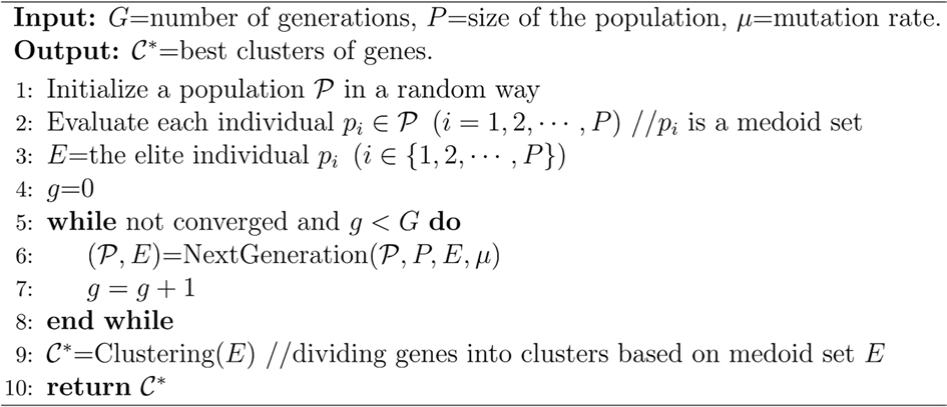
**Algorithm 1.** PAM-GA

- (1) Objective function (fitness function)

The objective function of this GA is the Silhouette Score. Suppose a gene *i* is assigned into a certain cluster, then the Silhouette Score of it is defined as follows:

2 $${s}_{i}=\frac{{b}_{i}-{a}_{i}}{max({a}_{i},{b}_{i})}$$

In the Formula 2, ${a}_{i}$ denotes the average distance between gene *i* and the genes in the same cluster, and ${b}_{i}$ denotes the average distance between gene *i* and the genes in other clusters. When the Silhouette Scores of all genes are calculated, the mean of the Silhouette Scores of clustering is calculated by the following formula:

3 $$S=\frac{1}{n}\sum_{i=1}^{n}{s}_{i}$$

where, *n* denotes the total number of genes. The larger the $S$, the better the clustering effect. We clustered genes according to the searched medoids set (assigned each gene into the cluster represented by the medoid closest to it), and then calculated Silhouette Score to obtain the value of objective function.

- (2) Coding and initialization

In PAM-GA, an individual is a medoid set in the clustering. To this end, each gene is numbered as an integer in the $$[0, n)$$. And an individual can be represented by a set $$I={\{x}_{1},{x}_{2},\cdots ,{x}_{k}\}$$*,* where $${x}_{i}$$ denotes the ID of the gene selected as the $${i}_{th}$$ medoid (to avoid confusion, $${x}_{i}$$ is called an element in this paper), and *k* denotes the number of clusters determined by PAM in Partitioning around medoids. In order to diversify the initial population, we used the initial individuals randomly produced, and the medoid set obtained by PAM was also included in the initial population. The size of the initial population ($$P$$) was set to 8000.

**Figure:**
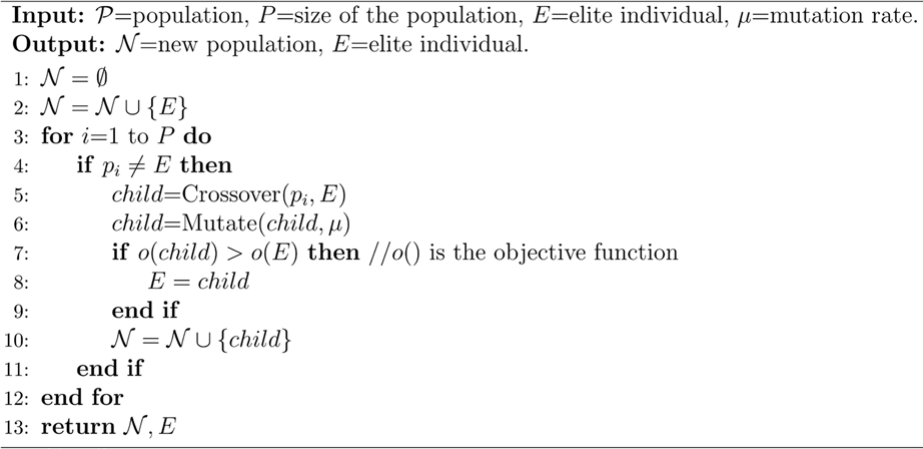
**Algorithm 2.** NextGeneration

- (3) Elite crossover strategy

Crossover and mutation operations are to generate the next generation of populations, as shown in Algorithm 2. To make the model converge quickly, we used the elite crossover strategy, which let all individuals in a generation cross with elite individual in turn, and elite individual could be replaced by one with better fitness at any time (Algorithm 2, line 3 ~ 12). This strategy is conducive to inheriting enough information from elite individuals, which enables the algorithm to explore more promising areas. During crossover, PAM-GA firstly merges all elements of the two parents, and then randomly selects *k* elements as children (Algorithm 3).

**Figure:**
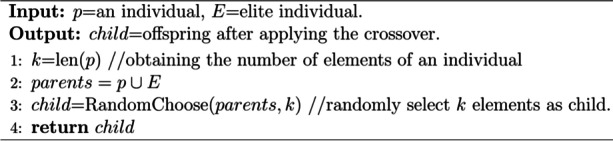
**Algorithm 3.** Crossover (PAM-GA)

- (4) Mutation

Mutation is the process of increasing diversity of the population and exploring new search region by introducing random variations in the individuals. The mutation operator of PAM-GA is as follows: an element in the individual is removed with a mutation probability (default was 0.1), and a gene not in this individual is randomly added. In particular, the elements in individual are guaranteed not to be duplicated after the mutation operation (Algorithm 4).

**Figure:**
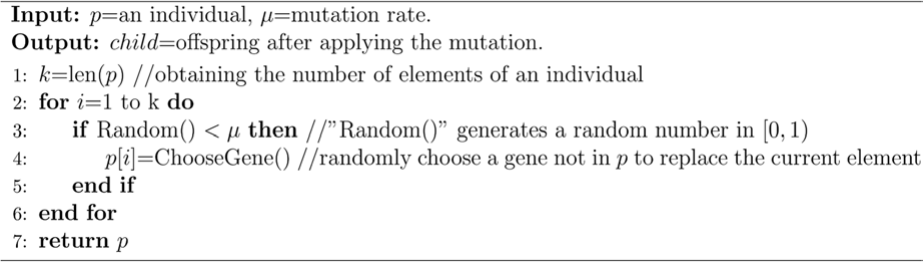
**Algorithm 4.** Mutation (PAM-GA)

- (5) Termination conditions

There are two termination conditions for PAM-GA, and the algorithm can be ended if anyone is satisfied: One is to reach the maximum number of iterations ($G$) ($G=20$ in PAM-GA). The other is that the fitness of elite individual has not changed in the three generations.

### Functional similarity optimization of gene modules

Inspired by NEO-K-Means [28], we designed a genetic algorithm (FSO-GA) to adjust the membership module for each gene, and considered both functional similarity and expression similarity during the adjustment. The operation process is as follows: Firstly, the adjustment rules of the membership modules for each gene are defined based on the initial modules (from the first stage). Then GA is used for combination optimization based on adjustment rules, and the optimization objective is the GO functional similarity within gene modules. Due to the complexity of gene functions in organisms, a gene often participates in multiple biological processes at the same time. So FSO-GA is an overlapping clustering algorithm, that is, a gene can belong to multiple modules in FSO-GA.

#### Adjustment rules of the membership modules for each gene

**Fig. 2:**
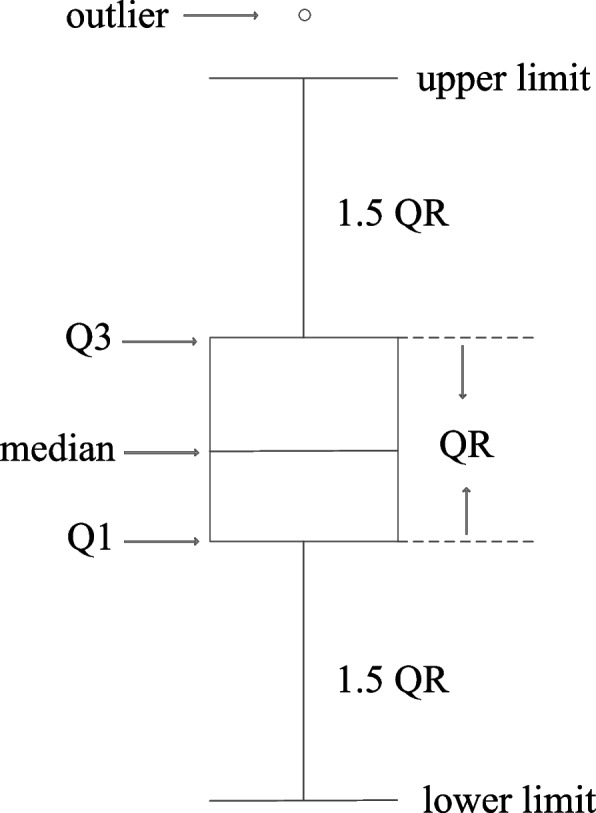
The structure of box plot

Generally, the distance between a gene and the center of a cluster can be used to determine whether the gene can be assigned into this module. In this work, if the distance (Formula 1) between a gene and a medoid (obtained by PAM-GA) was less than a threshold, it was considered that the gene could be assigned into the module represented by this medoid, so that a list of potential membership modules for each gene could be generated. The threshold is calculated as follows:

4 $$Threshold=Q3+1.5*(Q3-Q1)$$

where, $Q1$ and $Q3$ denote the lower and upper quartile of all distance from genes to medoid in a cluster, and $Q3-Q1$ is called the quartile range (QR). Formula 4 is the outlier cut-off point (upper limit) of the distance from intra-cluster genes to medoid (Fig. 2).

#### Genetic Algorithm for Functional Similarity Optimization (FSO-GA)

The overall process of this GA is similar to that of PAM-GA (for the pseudo-code, please refer to Algorithm 1 ~ 2). The elite individual obtained by FSO-GA is the result of functional similarity optimization.

- (1) Objective function (fitness function)

To calculate the functional similarity of a module, GO enrichment analysis was performed on genes within a module using the "enrichr ()" in "gseapy" python package. GO annotation files for human genes came from two databases: GeneOntology (http://geneontology.org/) and STRING (https://string-db.org/). We merged these two files to get the final GO annotation dataset (Supplementary Table S1). Since biological process (BP) was of great significance and was the most widely used category in GO, we only considered BP in this work (molecular function and cellular component could be calculated in the same way). The enriched BPs with Adjusted *P*-value < 0.05 were considered statistically significant, and these BPs were used for further analysis.

Next, we used the method proposed by Lin [29] to calculate the similarity of the BPs enriched by the same module. This method is an algorithm for similarity measurement of GO-terms based on information theory, which tries to maximize the similarity between two GO-terms which share more information. Therefore, the concept of IC is introduced: For any GO term $t$, $p(t)$ denotes the probability of finding a child of $t$ in the DAG. According to the information theory, the IC of $t$ can be expressed as $-log(p(t))$. Then the similarity of GO-terms ${t}_{i}$ and ${t}_{j}$ is obtained by the following formula:

5 $${Sim}_{lin}\left({t}_{i},{t}_{j}\right)=\frac{2\times {max}_{t\in S({t}_{i},{t}_{j})}[log(p(t))]}{log(p({t}_{i}))+log(p({t}_{j}))}$$

where $S({t}_{i},{t}_{j})$ denotes the set of parents shared by ${t}_{i}$ and ${t}_{j}$. Obviously, ${sim}_{lin}\left({t}_{i},{t}_{j}\right)\in \left[\mathrm{0,1}\right]$. In order to consider the similarity and significance of BPs in a module at the same time, the objective function is calculated based on Formula 6:

6 $$Target=Sim\times \alpha +Sig$$

where $\alpha$ is a weight coefficient, which is used to balance functional similarity and enrichment significance. $Sim$ is the functional similarity within the module, as shown in Formula 7 ~ 8. $Sig$ is the enrichment significance of the module, as shown in Formula 9.

7 $${Sim}_{i}=\frac{1}{num-1}\sum_{i\ne j}{Sim}_{lin}\left({t}_{i},{t}_{j}\right)$$

8 $$Sim=\frac{1}{sum}\sum_{i}^{sum}{Sim}_{i}$$

9 $$Sig=\frac{1}{k}\sum_{i}^{sum}-{\mathrm{log}}_{10}{P}_{i}$$

where ${t}_{i}$ and ${t}_{j}$ denote BP-terms, $num$ is the number of BPs involved in the calculation in the module to which ${t}_{i}$ belongs, ${Sim}_{i}$ denotes the similarity between the ${i}_{th}$ BP (${t}_{i}$) and other BPs (${t}_{*}$) in the same module, $sum$ is the number of BPs involved in the calculation of all modules, ${P}_{i}$ is the Adjusted *P*-value corresponding to ${t}_{i}$. Notably, a gene module might include a lot of BPs, but we usually only focused on some BPs with the highest significance, which represented the most important biological processes that the gene module was involved in. Therefore, we referred to the practice of most studies: If more than 20 BPs were in a module, only the 20 BPs with the highest enrichment significance (Adjusted *P*-value) would be studied. This operation also saved computing time.

- (2) Coding and initialization

Due to overlapping clustering, the result of FSO-GA can be represented by a matrix ${A}_{n\times k}$, where $n$ denotes the total number of genes in the dataset, and $k$ denotes the number of clusters (consistent with PAM-GA). Then any element ${a}_{i,j}$ in $A$ satisfies Formula 10, and an individual in the population can be represented by a matrix $A$.

10 $$a_{i,j}=\left\{\begin{array}{c}1,\;gene\;i\;belongs\;to\;module\;j\\0,\;gene\;i\;does\;not\;belong\;to\;module\;j\end{array}\right.$$

According to the list of potential membership modules for each gene, we randomly assigned a gene to 1~$M$ modules (the number was randomly generated), where *M* was the maximum number of modules this gene could belong to, and an individual was obtained after all genes were assigned. Moreover, to control the overlap rate between modules, we set a probability threshold *over*. If the random probability was less than *over*, the gene could belong to 2 or more modules (except genes with $M=1$); and if it was greater than *over*, the gene could only belong to one module (Algorithm 5, line 9 ~ 13). Of note, if $over=1$, it meant that all genes could be assigned into 1~$M$ modules (Algorithm 5, line 15). In particular, gene modules from PAM-GA were also included in the initial population (Algorithm 5, line 3).

**Figure:**
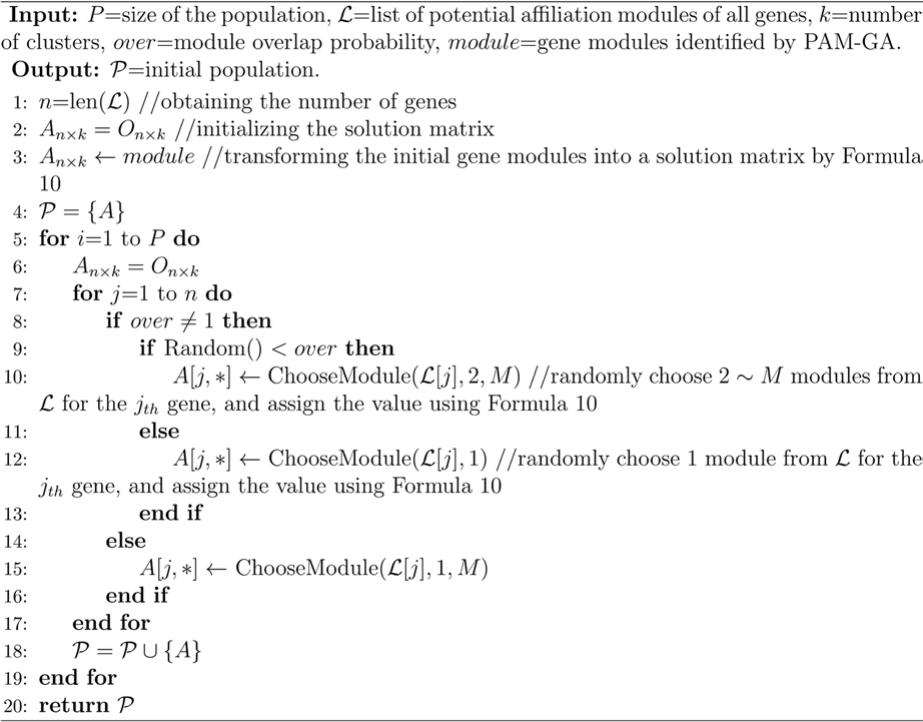
**Algorithm 5.** Initialization (FSO-GA)

- (3) Elite crossover strategy

Similar to PAM-GA, the elite crossover strategy is still applied to FSO-GA (Algorithm 2, line 3 ~ 12). During crossover, a truncation point $cut$(Algorithm 6, line 2) is randomly selected in $[1, n]$, and then the first $cut$ lines of one parent are merged with the $cut+1\sim n$ lines of another parent to form a child (Algorithm 6, line 8).

**Figure:**
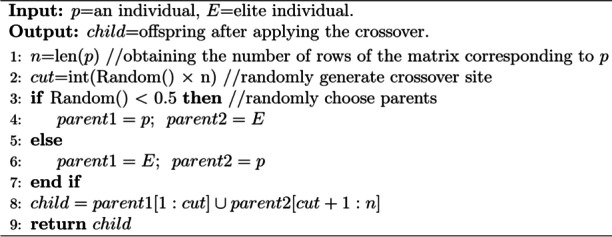
**Algorithm 6.** Crossover (FSO-GA)

- (4) Mutation

The mutation operation of FSO-GA is to reassign a gene to new modules with a certain mutation probability. Firstly, all ${a}_{i,*}$ corresponding to the gene $i$ are set to 0, then the modules to which gene $i$ belongs are redefined according to the method in the initialization (Algorithm 5, line 8 ~ 16).

- (5) Termination conditions

Termination conditions for FSO-GA are the same as for PAM-GA, see Improved Partitioning Around Medoids Based on Genetic Algorithm (PAM-GA) section  (5).

- (6) Parameter values

The parameter values used by FSO-GA are shown in Table 1. The values of *over* and α should be determined according to the actual situation, that is, different datasets have different values. We obtained the best values corresponding to each dataset after many experiments.

**Table 1:** Parameters of FSO-GA and values used in this work

Parameter	Description	Value
*P*	Size of the population	1000
*G*	Number of generations	20
$\mu$	Mutation rate	0.1
*over*	Module overlap probability	Determined by the dataset
$\alpha$	Weighting factor for balancing functional similarity with enrichment significance	Determined by the dataset

### Benchmark to compare the performance of GMIGAGO with existing methods

#### Data collection and preprocessing

To compare GMIGAGO with state-of-the-art methods, we tested 6 gene expression datasets (five RNA-seq datasets, and one microarray dataset). Three of them are from The Cancer Genome Atlas (TCGA, https://cancergenome.nih.gov) and three are from the Gene Expression Omnibus (GEO, https://www.ncbi.nlm.nih.gov/geo/).

- (1) TCGA datasets
- We obtained RNA-seq data of three cancers from TCGA: Breast Invasive Carcinoma (BRCA, 1080 samples), Thyroid Carcinoma (THCA, 500 samples), and Head and Neck Squamous Cell Carcinoma (HNSC, 432 samples). The gene expression levels in these three datasets were Fragments Per Kilobase Million (FPKM), and the preprocessing steps were as follows: ① Removing genes with lower expression. Genes whose expression levels were 0 in more than 20% of the samples were removed. Note that, if such genes do not exist in the data set, this step can be ignored. ② Differential expression analysis. We calculated the fold change of each gene's FPKM between cancer and normal tissues, and carried out t-test using the “ttest_ind()” in “scipy.stats” python package. Next, we retained genes (differentially expressed genes, DEGs) with Fold Change ≥ $x$ or Fold Change ≤ $1/x$ and *P*-value < 0.05, and the FPKMs of these genes in cancer samples were the final training dataset ($x=1.3$ in all TCGA datasets). Of note, the $x$ depends on the dataset which can be determined according to the traditional differential expression analysis process. The above two-steps are similar to the preprocessing of gene expression data in most studies. It not only removes noise genes in the dataset but also selects genes with research value, which ensures the reliability of subsequent analysis.
- (2) COVID-19 dataset
- The dataset is extracted from GEO (GSE161777), including RNA-seq data (read counts, 40 samples) of peripheral blood of 13 COVID-19 patients at 6 pseudotimes, and samples from 14 healthy donors. Firstly, we converted the read counts into FPKM, and removed the genes with lower expression. Then the differential expression analysis was performed for each pseudotime ($x=2.5$). Finally, the DEGs of all pseudotimes were merged, and the FPKMs of these genes in 40 patients were the final training dataset.
- (3) Stem dataset
- The dataset is extracted from GEO (GSE122380), including RNA-seq data (read counts) for 19 Yoruba individuals at 16 time points each during the differentiation from iPSC to cardiomyocyte (297 samples). Above all, read counts were converted into FPKM, and the genes with lower expression were removed. Further, the feature selection technique (random forest) was used to identify the top 3000 genes with the most significant expression changes at different time points. The FPKMs of these genes in 297 samples were taken as the final training dataset.
- (4) Radiation dataset
- The dataset is extracted from GEO (GSE161777), including microarray gene expression profiles of human peripheral blood exposed to 0 Gy (control), 0.15 Gy, 0.3 Gy, 1.5 Gy ionizing radiation for 1 h, 2 h, and 6 h. We only used the samples at 1 h. Differential expression analysis was performed between 0.15 Gy, 0.3 Gy, and 1.5 Gy with 0 Gy respectively ($x=1$), and all DEGs were merged as the final training dataset.

#### Evaluation indicators

We selected two evaluation indicators: Silhouette Score ($S$, calculated by Formula 3) was used to evaluate PAM-GA. Functional similarity within the module ($Sim$, calculated by Formula 8) was used to evaluate FSO-GA. It can be known from Formula 3 and 8 that $S\in [-1, 1]$, $Sim\in \left[0, 1\right]$, and the larger the two indicators, the better the performance of algorithms.

#### State-of-the-art algorithms selected for comparison

We compared PAM-GA with five clustering algorithms for the initial identification of gene modules on six datasets, which were all common clustering algorithms that accepted custom distance. The distance in Formula 1 was used in all algorithms. The five comparison algorithms and their parameter settings are as follows:

- (1) Density-Based Spatial Clustering of Applications with Noise (DBSCAN), using the “DBSCAN()” in “sklearn” python package. The parameter “min_samples” got value by steps of 1 in $$[2, 20)$$. The parameter “eps” got value by steps of 0.2 in $$[0.2, 4)$$. Finally, the clustering was performed using the combination of parameters that made Silhouette Score optimal.
- (2) Affinity Propagation, using the “AffinityPropagation()” in “sklearn” python package. Parameters were set to default values.
- (3) Hierarchical Clustering, using the “AgglomerativeClustering()” in “sklearn” python package. The parameter “n_clusters” got value by steps of 1 in $[8, 20]$. The parameter “linkage” got value in the set {“complete”, “average”, “single”}. Finally, the clustering was performed using the combination of parameters that made Silhouette Score optimal.
- (4) Spectral Clustering, using the “SpectralClustering()” in “sklearn” python package. The parameter “n_clusters” got value by steps of 1 in $[8, 20]$. The parameter “gamma” got value in the set $\{1\mathrm{E}-09, 1\mathrm{E}-08, 1\mathrm{E}-07, 1\mathrm{E}-06, 1\mathrm{E}-05, 1\mathrm{E}-04, 1\mathrm{E}-03\}$. Finally, the clustering was performed using the combination of parameters that made Silhouette Score optimal.
- (5) Partitioning Around Medoids (PAM). See Partitioning around medoids section for the experimental program.

Similarly, to compare the results of functional similarity optimization of gene modules, we performed FSO-GA and five state-of-the-art gene module identification algorithms on six datasets. And in order to have a fair comparison, we used the parameters recommended by the authors in all five algorithms. The details are as follows ((1), (2), (3) are non-overlapping gene module identification algorithms, and (4), (5) are overlapping gene module identification algorithms):

- (1) Weighted Gene Co-expression Network Analysis (WGCNA) [7, 8].
- (2) GCNA-Kpca [4].
- (3) k-module algorithm [13].
- (4) Overlapping CoExpressed gene Module (oCEM) [3].
- (5) NEO-PAM. This algorithm was an implementation of NEO-K-Means in our work, which replaced K-Means with PAM in NEO-K-Means, and other parts were consistent with NEO-K-Means. NEO-PAM expands the distance space of NEO-K-Means, so that it can be directly compared with FSO-GA.

### Application analysis

In order to better utilize GMIGAGO for the mining of life mechanisms, we designed a bioinformatics framework for subsequent analysis of the gene modules, and tried to explain the molecular mechanism of BRCA, THCA, HNSC, COVID-19, Stem, and Radiation to a certain extent. Firstly, the BPs enriched for each gene module (the output of GMIGAGO) were analyzed in detail to identify the module of interest. Then the module of interest was input into the STRING database to retrieve the protein interaction relationships (confidence score ≥ 0.400 was set as significant), and a protein–protein interaction (PPI) network was constructed. Finally, the degree of each node (gene) in the PPI network was calculated, and the genes with larger degree were defined as the hub genes.

## Results

### Results of initial identification of gene modules

DBSCAN, Affinity Propagation, Hierarchical Clustering, Spectral Clustering, PAM, and PAM-GA were applied to six datasets of BRCA, THCA, HNSC, COVID-19, Stem, and Radiation, respectively (Table 2). It can be seen that our PAM-GA shows the best clustering effect (the highest Silhouette Score) in the six datasets: 0.132069 (BRCA), 0.178543 (THCA), 0.132306 (HNSC), 0.216090 (COVID-19), 0.280704 (Stem), 0.142994 (Radiation). Notably, PAM-GA identifies 11 modules in BRCA, 10 modules in THCA, 8 modules in HNSC, 9 modules in COVID-19, 9 modules in Stem, and 9 modules in Radiation.

**Table 2:** The comparative results of different clustering algorithms (DBSCAN, Affinity Propagation, Hierarchical Clustering, Spectral clustering, PAM, and PAM-GA) on BRCA, THCA, HNSC, COVID-19, Stem, and Radiation datasets

Datasets	Algorithm	Silhouette Score	Parameter Value
BRCA	DBSCAN	0.010419	min_samples = 3, eps = 0.6
	Affinity Propagation	-0.470702	
	Hierarchical Clustering	0.097262	n_clusters = 8, linkage = ”average”
	Spectral Clustering	-0.331269	n_clusters = 8, gamma = 1E-04
	PAM	0.109636	k = 11
	PAM-GA	**0.132069**	k = 11
THCA	DBSCAN	0.037134	min_samples = 9, eps = 0.3
	Affinity Propagation	-0.558596	
	Hierarchical Clustering	0.137183	n_clusters = 8, linkage = ”average”
	Spectral Clustering	-0.381021	n_clusters = 8, gamma = 1E-05
	PAM	0.129793	k = 10
	PAM-GA	**0.178543**	k = 10
HNSC	DBSCAN	0.036295	min_samples = 11, eps = 0.5
	Affinity Propagation	-0.474879	
	Hierarchical Clustering	0.076271	n_clusters = 8, linkage = ”average”
	Spectral Clustering	-0.293960	n_clusters = 8, gamma = 1E-07
	PAM	0.109274	k = 8
	PAM-GA	**0.132306**	k = 8
COVID-19	DBSCAN	0.064597	min_samples = 13, eps = 0.2
	Affinity Propagation	-0.593570	
	Hierarchical Clustering	0.128473	n_clusters = 18, linkage = ”average”
	Spectral Clustering	-0.431040	n_clusters = 8, gamma = 1E-06
	PAM	0.177675	k = 9
	PAM-GA	**0.216090**	k = 9
Stem	DBSCAN	0.161855	min_samples = 12, eps = 0.2
	Affinity Propagation	-0.698527	
	Hierarchical Clustering	0.167391	n_clusters = 9, linkage = ”average”
	Spectral Clustering	-0.525442	n_clusters = 8, gamma = 1E-03
	PAM	0.239311	k = 9
	PAM-GA	**0.280704**	k = 9
Radiation	DBSCAN	0.023345	min_samples = 4, eps = 0.3
	Affinity Propagation	-0.564850	
	Hierarchical Clustering	0.080373	n_clusters = 8, linkage = ”average”
	Spectral Clustering	-0.372413	n_clusters = 8, gamma = 1E-06
	PAM	0.092876	k = 9
	PAM-GA	**0.142994**	k = 9

^*^The last column denotes the optimal parameter values obtained after many experiments

### Results of functional similarity optimization of gene modules

**Fig. 3:**
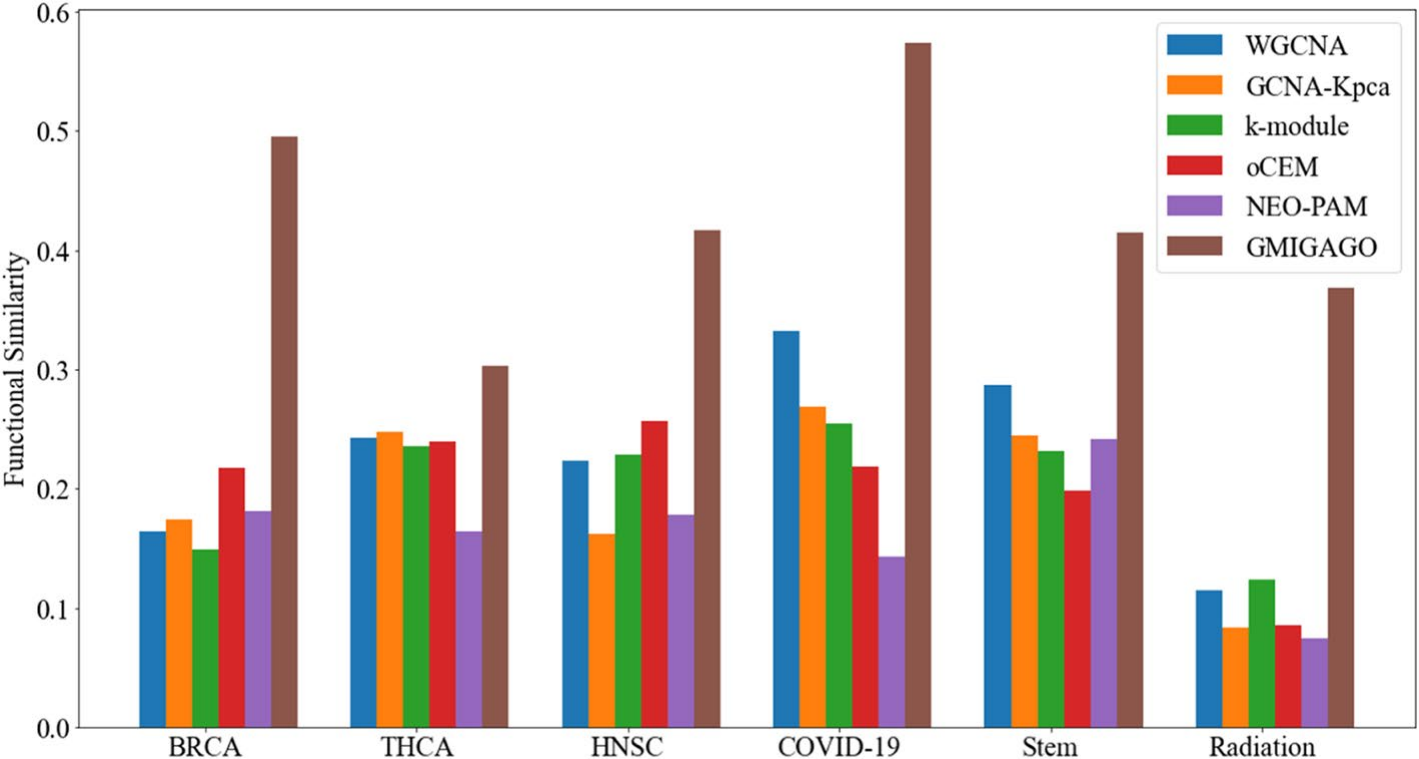
The comparative results of different module identification algorithms (WGCNA, GCNA-Kpca, k-module, oCEM, and NEO-PAM) on BRCA, THCA, HNSC, COVID-19, Stem, and Radiation datasets

Taking the initial gene modules (obtained by PAM-GA) as input, the functional similarity within gene modules was optimized by FSO-GA (GMIGAGO). Compared with WGCNA, GCNA-Kpca, k-module, oCEM, and NEO-PAM, the functional similarity in the six datasets is shown in Fig. 3.

The functional similarity of the results obtained by our GMIGAGO is significantly better than that of the comparison algorithms in all datasets: 0.495307 (BRCA), 0.303148 (THCA), 0.416422 (HNSC), 0.573636 (COVID-19), 0.414609 (Stem), 0.368670 (Radiation). The modules obtained by GMIGAGO of the six datasets (i.e. matrix $A$, elements defined by Formula 10) are shown in Supplementary Table S2.

After many experiments, the values of the parameter *over* are: 1 (BRCA), 1 (THCA), 1 (HNSC), 0.3 (COVID-19), 0.3 (Stem), 1 (Radiation); the values of the parameter $\alpha$ are: 1800 (BRCA), 1800 (THCA), 1800 (HNSC), 1000 (COVID-19), 1800 (Stem), 1000 (Radiation).

### Application analysis

#### Gene modules of BRCA

**Fig. 4:**
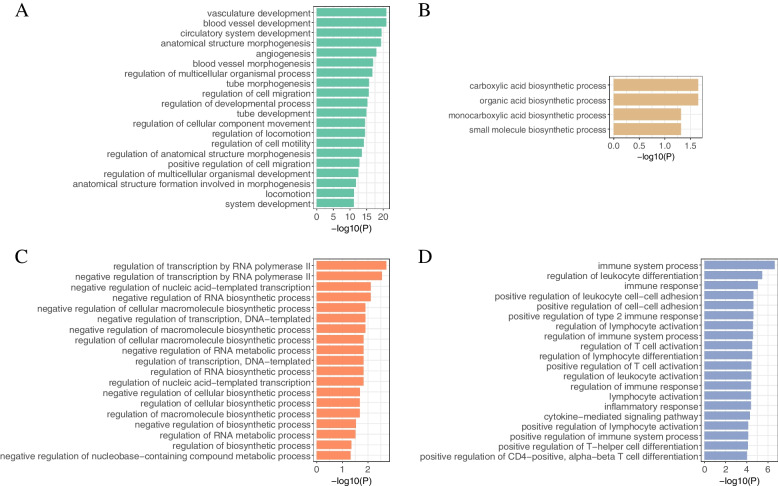
The GO Terms (biological processes) in module A ~ module D of BRCA. *Y-axis denotes BPs, and X-axis denotes the negative logarithm of P-value*

**Fig. 5:**
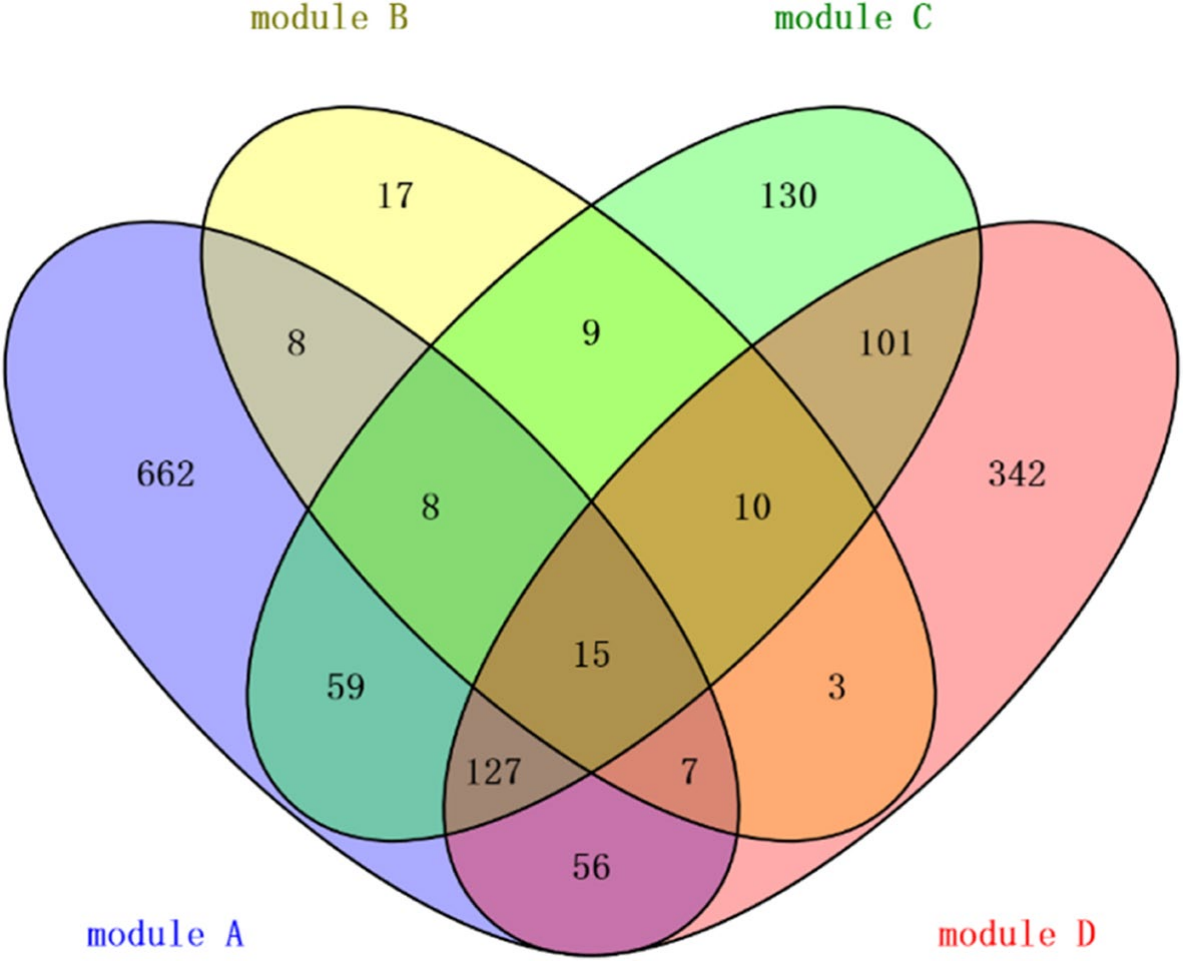
Venn diagrams of genes in module A ~ module D in BRCA

Taking BRCA as an example, we tried to analyze its molecular mechanism from functional gene modules to show the downstream application of GMIGAGO. As mentioned above, 11 gene modules were identified in BRCA by GMIGAGO, and the results were shown in Supplementary Table S2. Because there were too many modules, we selected four of them for detailed analysis. The BPs of these modules are shown in Fig. 4 (Other modules are mainly related to mitosis and cell cycle). Module A is involved in BPs related to development, module B is involved in BPs related to biosynthesis, module C is involved in BPs related to the regulation of nucleic acid metabolism, and module D is involved in BPs related to immunity. Due to overlapping clusters, the modules have both the same genes and their own specific genes, which reflects the complexity of gene functions (Fig. 5).

As we all know, cancer cells are characterized by self-sufficiency in the absence of growth signals, their ability to evade apoptosis, resistance to anti-growth signals, sustained angiogenesis, uncontrolled proliferation, and invasion and metastasis [30]. On the other hand, metabolic reprogramming, including enhanced biosynthesis of macromolecules, altered energy metabolism, and maintenance of redox homeostasis, is considered a hallmark of cancer, sustaining cancer cell growth [31]. Willmann et al. believed that the altered RNA metabolism of cancer cells resulted in elevated excretion levels of modified nucleosides in different biological fluids, and modified nucleosides had great potential as biomarkers of breast cancer [32]. In addition, the polyamine biosynthetic pathway and the hexosamine biosynthetic pathway play a key role in the growth of breast cancer cells and are closely related to the prognosis of breast cancer [33, 34]_._ Finally, human immunity, especially cellular immunity, is closely related to the occurrence and development of tumors. And the presence of tumor-specific immune responses in breast cancer has been suppressed, even in the early stages of cancer development [35]. To sum up, these gene modules identified by GMIGAGO play important roles in the occurrence and development of breast cancer.

#### Analysis of immunity module in BRCA

**Fig.6:**
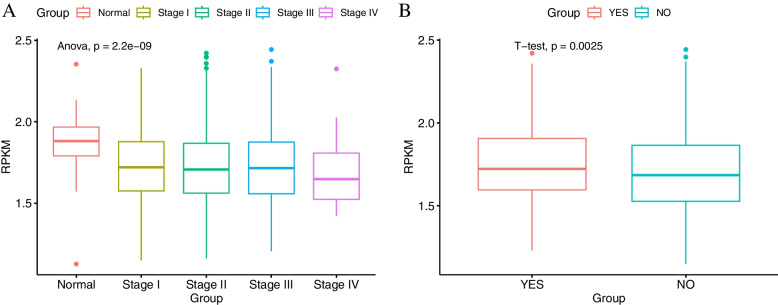
Association analysis of immunity module and clinical indicators in BRCA. **A** Cancer progression stage. **B** immune response after treatment. *A point denotes the median expression of immunity module in a sample*

Cancer is closely related to immune processes, and immunity is suppressed in BRCA [35]. Therefore, we focused on immunity module D, and analyzed its association with two clinical indicators (cancer progression stage, immune response after treatment) using T-test and Anova (Fig. 6). The immunity module is significantly down-regulated from Stage I to Stage IV of BRCA ($P-\mathrm{value}=2.2\mathrm{E}-09$), especially the lowest expression level in Stage IV, which supports the views of reference [35] (Fig. 6A). Moreover, module D in patients with immune response (after treatment) is more up-regulated than that in patients without immune response ($P-\mathrm{value}=0.0025$, Fig. 6B), suggesting that this module can be used as potential biomarkers for immune response after treatment in BRCA.

**Fig. 7:**
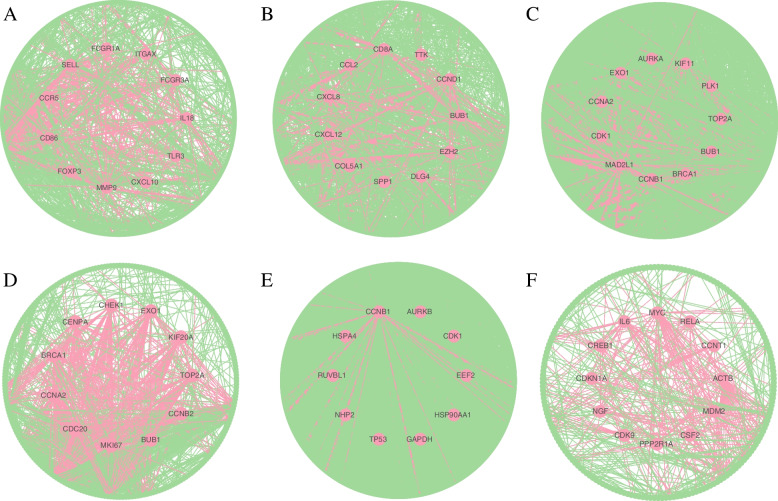
PPI networks of key modules. **A** BRCA. **B** THCA. **C** HNSC. **D** COVID-19. **E** Stem. **F** Radiation. *The nodes denote the genes, the edges denote the protein interaction relationships, and the red nodes denote hub genes*

Next, based on these gene modules, we identified hub genes in BRCA that were involved in specific biological processes (the immune process was taken as an example in this work). The PPI network formed by the immunity module is shown in Fig. 7A, which includes 467 genes. We defined the 11 genes with the largest *degrees* as hub genes (the *degrees* of the 9th, 10th, and 11th genes were equal), and these genes were arranged in descending order of *degrees* as follows: CD86, FCGR3A, FOXP3, ITGAX, CXCL10, MMP9, CCR5, SELL, FCGR1A, TLR3, IL18. Notably, by querying the DisGeNET database (https://www.disgenet.org/) (the results were in Supplementary Table S3), these genes were associated with malignant tumors (especially breast cancer).

The percentage of CD86 ( +) B cells showed a positive relationship with higher tumor grade and higher numbers of involved lymph nodes in 44 breast cancer patients [36]. FCGR3A encodes a receptor for the Fc portion of immunoglobulin G, which plays a significant role in the immune response [37]. And FCGR3A has potential clinical relevance in the treatment of breast cancer [38]. FoxP3 + regulatory T cells (Tregs) can suppress antitumor immunity, and the ratio of CD8 + to FoxP3 + cells may be predictive for the clinical outcome of medullary breast cancer [39]. CD11c (ITGAX)-targeted protein vaccines for in vivo delivery of tumor antigens to DCs induce potent immune responses and antitumoral activities [40]. CXCL10 acts on CD4 + and CD8 + T cells to enhance antitumor immunity, and the serum level of CXCL10 in breast cancer patients is positively correlated with tumor size and ER status [41, 42]. MMP9 is associated with inflammation and inhibits platelet aggregation [43]. The early metastatic circuit can be disrupted by inhibiting active MMP9, and warrant further studies of MMP9-targeted anti-metastatic breast cancer therapy [44]. The 32 bp deletion in CCR5 may be associated with breast cancer, and the malformed CCR5 receptor may not be able to express and function normally, thereby reducing immunity to cancer and leading to cancer progression [45]. Gene Set Enrichment Analysis (GSEA) revealed the association of SELL expression with several immunological features in breast cancer [46]. FCGR1A is associated with immune infiltration levels in various cancers [47], and it’s correlated with recurrence-free survival in patients with triple-negative breast cancer (TNBC) [48]. TLR3 is very important in immune escape in breast cancer [49]. Interleukin-18 (IL-18) is a key cytokine responsible for immune response and involves in the process of cancer development. The IL18-607 and IL18-137 polymorphism contribute to increase the breast cancer risk [50]. In summary, the 11 immunity-related HUB genes in BRCA play key roles in the occurrence and development of breast cancer and the immune response of tumors, further proving that the gene modules identified by GMIGAGO have good performance in mining the immunological regulation mechanism of breast cancer. These genes may be potential targets for immunotherapy in breast cancer.

#### Gene modules of other datasets

To further illustrate the role of GMIGAGO in gene module identification, we analyzed a key module in THCA, HNSC, COVID-19, Stem, and Radiation respectively.

**Fig. 8:**
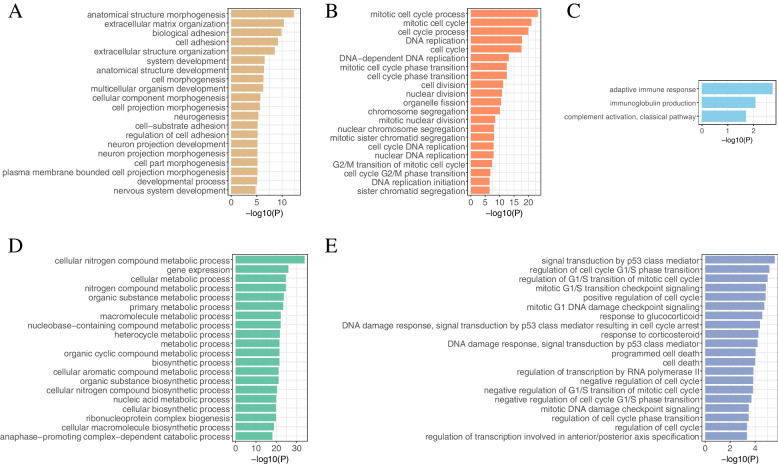
The GO Terms (biological processes) in key modules. **A** THCA. **B** HNSC. **C** COVID-19. **D** Stem. **E** Radiation. *Y-axis denotes BPs, and X-axis denotes the negative logarithm of P-value*

- (1) THCA
- We identified a development module in THCA, which was mainly involved in development-related BPs such as anatomical structure morphogenesis, system development, and anatomical structure development (Fig. 8A). Most cancers result in developmental disorders, which are important hallmarks of cancers [30]. The PPI network formed by this module is shown in Fig. 7B. We defined the 11 genes with the largest *degrees* as hub genes in this module (the *degrees* of the 10th, and 11th genes were equal), which were arranged in descending order of *degrees* as follows: CD8A, CCND1, EZH2, CXCL8, SPP1, DLG4, TTK, CXCL12, COL5A1, BUB1, CCL2. By querying the DisGeNET database (Supplementary Table S3), these genes were all associated with malignant tumors (especially thyroid cancer).

- (2) HNSC
- A cell cycle module was identified in HNSC, which was mainly involved in cell cycle-related BPs such as mitotic cell cycle, DNA replication, and nuclear chromosome segregation (Fig. 8B). Cancer is a group of diseases in which cells divide continuously and excessively, and cancer-associated mutations that perturb cell cycle control allow continuous cell division chiefly by compromising the ability of cells to exit the cell cycle [51]. The PPI network formed by this module is shown in Fig. 7C. We defined the 11 genes with the largest *degrees* as hub genes (the *degrees* of the 10th, and 11th genes were equal), and these genes were arranged in descending order of *degrees* as follows: CDK1, PLK1, CCNA2, CCNB1, KIF11, TOP2A, BUB1, BRCA1, MAD2L1, AURKA, EXO1. By querying the DisGeNET database (Supplementary Table S3), these genes were all associated with squamous cell carcinoma (especially HNSC).

- (3) COVID-19
- An immunity module was identified in COVID-19, which was mainly involved in immunity-related BPs such as adaptive immune response, immunoglobulin production, and complement activation, classical pathway (Fig. 8C). Researchers have found that the invasion of SARS-CoV-2 causes multiple organ dysfunction through immune system disorders and cytokine storm syndrome [52]. The PPI network formed by this module is shown in Fig. 7D. We defined the 11 genes with the largest *degrees* as hub genes (the *degrees* of the 10th, and 11th genes were equal), and these genes were arranged in descending order of *degrees* as follows: CCNA2, BRCA1, CHEK1, KIF20A, CDC20, EXO1, CENPA, BUB1, CCNB2, TOP2A, MKI67. By querying the DisGeNET database (Supplementary Table S3), these genes were all associated with virus diseases.

- (4) Stem
- A metabolism module was identified in Stem, which was mainly involved in metabolism-related BPs such as organic substance metabolic process, biosynthetic process, and nucleic acid metabolic process (Fig. 8D). Stem cell activation and differentiation occur along changes in cellular metabolism. Metabolic transitions translate into changes in redox balance, cell signalling and epigenetics, thereby regulating these processes [53]. The PPI network formed by this module is shown in Fig. 7E. We defined the 10 genes with the largest *degrees* as hub genes, and these genes were arranged in descending order of *degrees* as follows: TP53, GAPDH, HSP90AA1, CDK1, CCNB1, EEF2, HSPA4, NHP2, AURKB, RUVBL1.

- (5) Radiation
- A cell cycle module was identified in Radiation, which was mainly involved in mitotic G1/S transition checkpoint signaling, regulation of G1/S transition of mitotic cell cycle, positive regulation of cell cycle, and other BPs related to cell cycle, as well as mitotic G1 DNA damage checkpoint signaling, DNA damage response, signal transduction by p53 class mediator, and other BPs related to DNA damage (Fig. 8E). Ionizing radiation (IR)-induced DNA damage activates a number of DNA damage response and repair (DRR) signaling cascades that control cell cycle arrest, DNA repair, and the cell’s fate [54]. The PPI network formed by this module is shown in Fig. 7F. We defined the 12 genes with the largest *degrees* as hub genes (the *degrees* of the 10th, 11th, and 12th genes were equal), and these genes were arranged in descending order of *degrees* as follows: MYC, ACTB, IL6, RELA, MDM2, CREB1, CDK9, CCNT1, PPP2R1A, CDKN1A, NGF, CSF2. By querying the DisGeNET database (Supplementary Table S3), these genes were all associated with malignant tumors. Some studies have shown that IR is associated with a higher risk of cancer, with 10% of all cancers caused by IR above baseline [55].
- In summary, the gene modules identified by GMIGAGO perform important biological functions, and the hub genes can be used as potential targets for diseases or radiation protection, which further proves that GMIGAGO has excellent performance in mining molecular mechanisms.

## Discussion

Traditional gene co-expression module identification algorithms only consider the similarity of gene expression, while the functional similarity within the module is low. To solve this problem, we proposed a functional gene module identification algorithm based on genetic algorithm and gene ontology: GMIGAGO. This algorithm performs a two-stage clustering of genes: In the first stage, PAM-GA is used for the initial clustering of gene expression data (only considering the similarity of gene expression levels), and co-expression modules are obtained (to make the gene expression patterns within a module similar, we only considered the positive correlation degree of gene expression, and the negative correlation could also be considered in other tasks). In the second stage, based on the initial identification results, with the goal of improving the functional similarity within gene modules, FSO-GA is used to adjust the module to which the gene belongs. Compared with the other algorithms, the results obtained by PAM-GA had the highest Silhouette Score on all datasets, indicating that PAM-GA could identify better gene co-expression modules. Moreover, the functional gene modules adjusted by FSO-GA had the highest functional similarity (much higher than state-of-the-art algorithms). GMIGAGO considers both the expression similarity and function similarity within the modules at the same time, and can obtain gene modules with higher biological significance from the perspective of molecular biology.

NEO-K-Means [28] is an overlapping clustering algorithm proposed by Whang et al. It extends the objective function of K-Means and solves the overlapping and non-exhaustive problems uniformly. For a dataset containing $n$ samples, the algorithm does the following: (1) Select $k$ cluster centers arbitrarily, where $k$ is the number of clusters. (2) Assign $n-\beta n$ samples to the nearest cluster centers. (3) Sort the distances from all samples to all cluster centers, select the $\alpha n+\beta n$ smallest distances, and assign the samples to the corresponding clusters (except for assignments that already exist in (2)). (4) Take the mean of the samples in each cluster to get the new cluster center. (5) Go to (2) until the termination condition is met. To obtain gene modules with similar expressions and similar functions, we extended it based on the above ideas: Since PAM-GA had obtained better cluster centers and assignments based on the expression similarity, FSO-GA actually only needed to simulate one iteration in NEO-K-Means. Through the ingenious design of the objective function (Formula 6), FSO-GA can continuously improve the functional similarity and enrichment significance within the module in this process. Furthermore, unlike NEO-K-Means, we didn’t specify that a gene must belong to a certain module, but developed a list of potential membership modules for each gene through the rules of expression similarity. By the search of GA, the membership module for each gene was determined according to the objective function. This change lead more information of each gene to be considered, which was beneficial for obtaining gene modules with higher functional similarity. Data analysis showed that the modules obtained by our algorithm were better than that of NEO-K-Means.

There are many parameters in GMIGAGO, most of which come from GA (size of the population, number of generations, mutation rate, etc.), and their setting strategy is consistent with traditional GA, which can be adjusted according to actual problems. As FSO-GA needs to perform many GO enrichment calculations when obtaining individual fitness, and the time complexity is high, so the size of the population ($P$) and number of the generations ($G$) of FSO-GA should not be set too large. In general, we recommend that $P$ be set to 1000 ~ 2000, and $G$ be set to within 20 (these two values can be higher if there is enough computing power). In fact, after many experiments, it could be seen that FSO-GA had a fast convergence speed due to the elite crossover strategy (generally, it can converge after about 10 iterations), so there was no need to set these two parameters too large. On the other hand, GMIGAGO focuses on the appropriate optimization of functional similarity based on gene co-expression modules, rather than finding the global optimal solution (a group of optimal modules) with the highest functional similarity, so the cost of calculation is acceptable.

In particular, FSO-GA is sensitive to $over$ and $\alpha$. For *over*, it adjusts the overlap degree between modules (similar to the effect of $\alpha$ in NEO-K-Means). The larger the *over*, the more overlapping genes between modules, and the more modules with the same functions. Notably, due to genes performing the same functions might have different gene expression patterns, we allowed multiple modules to perform the same biological functions (for example, multiple modules in BRCA are mainly involved in cell cycle-related BPs, which are the most important BPs in BRCA). Generally, the $over$ can be set to 1 firstly. If there are too many modules with the same functions (the functions between modules cannot be distinguished), the *Over* can be gradually lowered to obtain better results. In addition, to maintain higher enrichment significance while increasing the functional similarity within the module, so as not to generate many modules with lower biological significance, we considered both functional similarity (*Sim*) and functional significance ($Sig$) in the objective function of FSO-GA and used parameter $\alpha$ to balance. The larger the $\alpha$ is, the larger the proportion of functional similarity in the objective function is. We aimed to maximize functional similarity within modules, so a larger $\alpha$ was used in this work. In practice, the value of $\alpha$ can be adjusted according to the actual situation.

As the potential data distribution is unknown, the number of clustering clusters (*k*) cannot be obtained by a perfect method at present [56]. Because a higher Silhouette Score represents a better clustering result, it is used to determine the number of clusters in many tasks [57]. Based on this, the prior knowledge and Silhouette Score were combined to determine the number of gene modules in our work. Namely, let *k* take values in $[8, 20]$ in turn and execute the clustering algorithm, and select the *k* that made Silhouette Score the highest in this range, where, the determination of the interval $[8, 20]$ is based on prior knowledge: the number of gene modules should not be too large or too small. Too few modules will lead to too many kinds of biological processes involved in a module, which is also not conducive to the optimization of gene modules; and too many modules will lead to a lot of invalid modules with few genes.

We took BRCA as an example to show the downstream application of GMIGAGO, including enrichment results analysis, association analysis between modules and clinical indicators, and identification of hub genes, etc. From the experimental results, the gene modules identified by GMIGAGO played important roles in BRCA, where the immunity module was down-regulated in all stages of BRCA, especially in advanced stages, and it also reflected the immune response of different patients after treatment. Therefore, GMIGAGO can characterize the occurrence and development of diseases at the molecular level, and it can also analyze the mechanism of individual differences. Moreover, the hub genes in the immunity module can be used as potential therapeutic targets. Furthermore, the results obtained by GMIGAGO can also be used to predict gene functions. Since the genes within a module have both expression similarity and functional similarity, these genes can be considered being related to a specific function. Taking the immunity module in BRCA as an example, we input the genes in this module into the immune database ImmPort [58] (https://www.immport.org) for query, and found that 71 genes (15.2% of the total number of genes in the module) existed in the ImmPort. Obviously, the immunity-related genes contained in ImmPort may not be complete, and the confirmed immunity-related genes in this module should be more than 71. On the one hand, these results prove the reliability of our functional gene modules. And on the other hand, it also shows that there are many genes in this module that have not been experimentally confirmed to be related to immunity, which can be predicted as genes involved in immune response in BRCA, laying a foundation for further screening biomarkers of breast cancer.

## Conclusion

In order to consider both expression similarity and functional similarity during gene module identification, GMIGAGO (a functional Gene Module Identification algorithm based on Genetic Algorithm and Gene Ontology) was proposed in this work. We showed that GMIGAGO identified the gene modules with the highest functional similarity (much higher than state-of-the-art algorithms). Through application analysis, it can be seen that GMIGAGO has excellent performance in mining molecular mechanisms.

## Supplementary Information

**Additional file 1: Table S1. **GO annotation dataset. GO annotation information from GeneOntology and STRING. The first column contains genes, the second column contains GO terms, and one row denotes an annotation relationship.

**Additional file 2: Table S2.** The modules obtained by GMIGAGO of the six datasets. A sheet contains the result of a dataset. Rows denote genes, columns denote modules, and elements satisfy Formula 10.

**Additional file 3: Table S3.** Query results of hub genes in DisGeNET database. A sheet corresponds to a hub gene.

## Data Availability

The raw RNA-Seq and microarray data used in this study have been deposited in the The Cancer Genome Atlas (TCGA, https://cancergenome.nih.gov) and Gene Expression Omnibus (GEO, https://www.ncbi.nlm.nih.gov/geo/) under the accession number GSE161777, GSE122380, and GSE161777.
